# Three-Dimensional Laparoscopic Nephrectomy for Benign Nonfunctioning Kidneys: A Single-Center Initial Experience

**DOI:** 10.7759/cureus.60352

**Published:** 2024-05-15

**Authors:** Ngoc Hung Pham, Khac Sang Phan, Cong Le Kha Bui, Van Quoc Anh Nguyen, Luong Vinh Le, Thanh Liem Ngo, Kim Tuan Nguyen, Van Can Truong, Ngoc Khanh Tran

**Affiliations:** 1 Department of Urology, Hue Central Hospital, Hue, VNM; 2 Department of Surgery, Hoan My Hospital, Binh Phuoc, VNM; 3 Deparment of Urology, Hue Central Hospital, Hue, VNM; 4 Deparment of General Surgery, Hue Central Hospital, Hue, VNM; 5 Department of General Surgery, Hue Central Hospital, Hue, VNM

**Keywords:** benign nonfunctioning kidney, laparoscopic nephrectomy, surgical outcome, benign, 3d laparoscopic nephrectomy, nonfunctioning kidney

## Abstract

Background: There are several types of benign renal diseases, such as urological stones, ureteropelvic junction obstruction, renal vascular disease, and inflammation, which are responsible for nonfunctioning kidneys. Laparoscopic nephrectomy (LN) is the gold standard for treating nonfunctioning kidneys with complications. This study presents the results of our initial experiences with 3D laparoscopic nephrectomy (3D-LN) for benign, nonfunctioning kidneys.

Methods: From July 2021 to July 2023, 40 consecutive patients who underwent 3D transperitoneal laparoscopic nephrectomy were retrospectively evaluated at the Department of Urology and Department of General Surgery, Hue Central Hospital, Hue, Vietnam. Patient demographics, intraoperative and early postoperative results, postoperative recovery, complications, and three-month follow-up results were recorded.

Results: The mean age was 58.35 ± 14.9 years. There were 13 (32.5%) male and 27 (67.5%) female patients. Flank pain was the main reason for hospitalization in 33 cases (82.5%); the common cause of a nonfunctioning kidney was urological stones (62.5%). Twenty-three out of 40 patients underwent a left nephrectomy. The average operative time was 92.57 ± 28.69 minutes. A statistically significant difference in surgery time was found between the group with no adhesion and the group with mild adhesion, as well as between the first 19 patients and the last 18 patients (p <0.05). The mean blood loss was 51.62 ± 24.35 ml. Three cases were converted to open surgery due to severe adhesions. The postoperative complications rate was 8.1%. The average length of the postoperative hospital stay was 7.89 ± 3.59 days.

Conclusions: Three-dimensional laparoscopic nephrectomy is a safe and effective method that increases depth perception and spatial orientation for surgeons and can compensate for the remaining shortcomings of traditional 2D systems.

## Introduction

Nonfunctioning kidneys can be a consequence of various diseases, such as urological lithiasis, ureteropelvic junction obstruction, renal tuberculosis, congenital disorders, and postoperative ureteral obstruction [1]. Renal resection is the most effective treatment for benign, nonfunctioning kidneys that present with complications such as abscesses, hypertension, and infectious pyelonephritis. Since Clayman et al. conducted the first laparoscopic nephrectomy (LN) [2], this minimally invasive technique has become a prevalent surgical option for the urologist in the treatment of benign, nonfunctioning kidneys. Moreover, it has a lot of advantages, such as less intraoperative blood loss, relief of postoperative pain, good cosmetics, a shorter hospital stay, and faster recovery time in comparison with open surgery [3].

However, contemporary laparoscopic systems are predominantly 2D imaging, where the primary limitation is poor depth perception during surgery [4]. Surgeons tackle this shortfall thanks to their experience and ability to visualize in depth. Consequently, the introduction of 3D laparoscopic surgical systems emerged in the early 1990s as a solution aimed at overcoming these limitations [5]. The 3D system provides accurate depth and estimation of anatomical distances in each dimension, thereby enhancing the dexterity of surgeons, accuracy, and speed of dissection, as well as a shorter learning curve [6]. Three-dimensional laparoscopic surgery compared to 2D surgery helps not only reduce surgery time and blood loss but also makes the surgeon more comfortable during surgery [7].

Despite the great benefits, 3D imaging systems have not been widely applied. At Hue Central Hospital, Hue, Vietnam, 3D laparoscopic surgery was first applied in 2015, then invested in and deployed regularly in 2017. Since 2020, we have applied 3D systems to surgery for urological diseases. Accordingly, the objective of the present study is to evaluate the intraoperative and early postoperative results of 3D laparoscopic nephrectomy (3D-LN) for benign nonfunctioning kidneys.

## Materials and methods

Patients

In this study, clinical data were obtained from a retrospectively maintained database approved by the review board and ethics committee of Hue Central Hospital, Hue, Vietnam. In our study of human subjects, we followed the ethical principles delineated in the 1964 Helsinki Declaration. We identified 40 patients at Hue Central Hospital who underwent 3D-LN for benign nonfunctioning kidneys from July 2021 to July 2023 based on the following indications: (1) Cases where the contralateral kidney function was ≥60 ml/min/1.73m^2^, the glomerular filtration rate (GFR) is <10 ml/min/1.73m^2^ [8] or renal hypofunction (on renal scintigraphy) is <10% [9]. (2) If the contralateral kidney function was <60 ml/min/1.73 m^2^, the kidney loses complete function (GFR is 0 ml/min/1.73m^2^). (3) Benign nonfunctioning kidneys with complications such as chronic urinary tract infection, hematuria, hypertension, chronic back pain, and compression of the surrounding organs.

**Figure 1:**
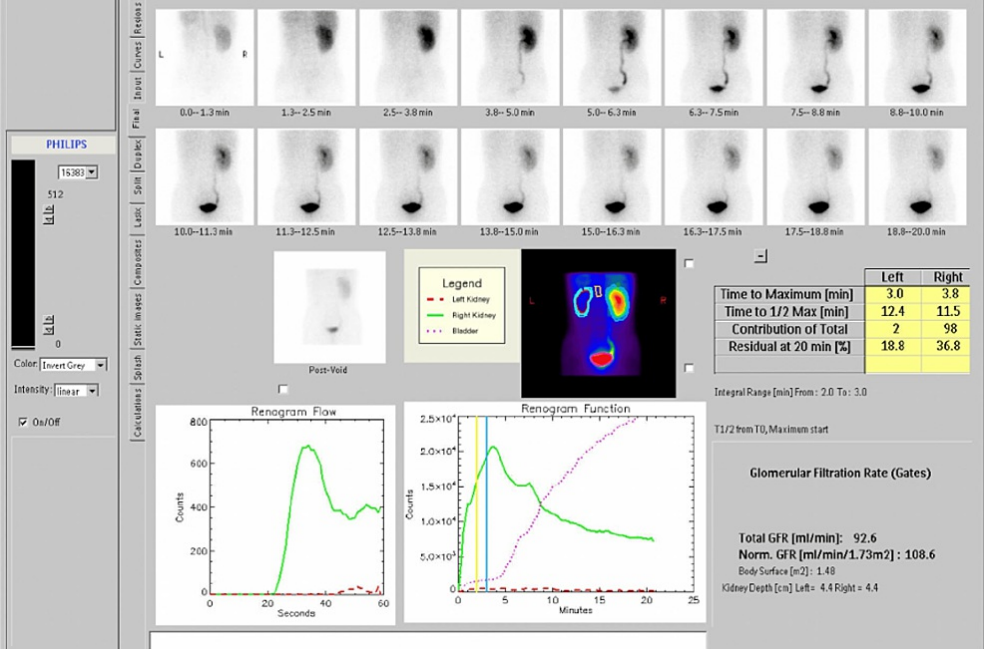
The Tc-99m DTPA renal dynamic scintigraphy showed complete loss of left kidney function. DTPA: diethylene-triamine-pentaacetate

**Figure 2:**
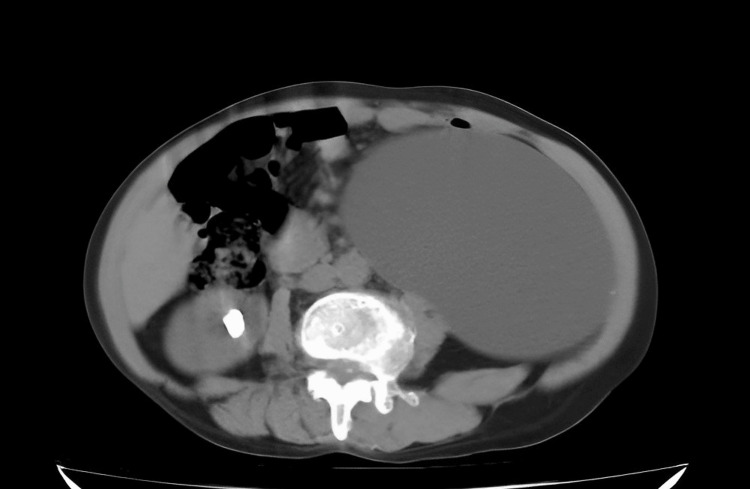
The CT scan image showed the left kidney with hydronephrosis and thin, dilated parenchyma.

We excluded cases with unstable urinary infections or malignant lesions. Each patient underwent ultrasonography and Tc-99m diethylene-triamine-pentaacetate (DTPA) renal dynamic scintigraphy (Figure 1) for evaluation and diagnosis, and the factors were confirmed using computerized tomography (CT) (Figure 2).

Techniques

All cases were performed via transperitoneal approach with the assistance of the 3D laparoscopic surgery system (Karl Storz SE & Co., Tuttlingen, Germany). We used three ports for the left side and four ports for the right side.

**Figure 3:**
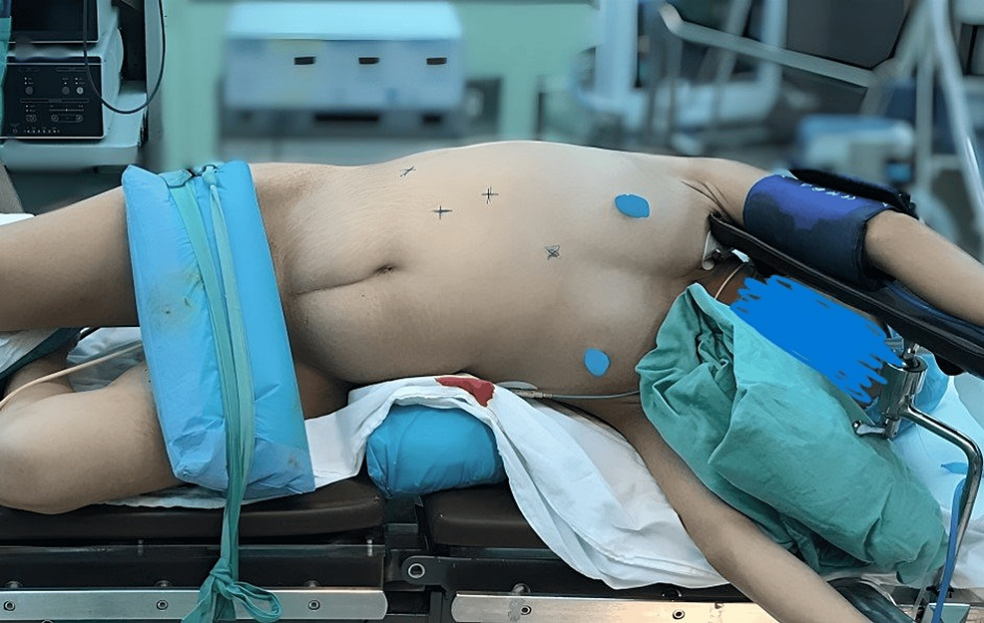
Patient position and trocar placement in cases of right nonfunctioning nephrectomy

The patient was positioned in a modified flank orientation at an angle of 45° to 60° (Figure 3). The pneumoperitoneum was established by inserting a Veress needle laterally into the umbilicus, followed by the placement of a 10 mm camera port. In cases involving the right side, a 12 mm secondary port was inserted between the anterior superior iliac spine and umbilicus. Additionally, a third port measuring 5 mm in diameter was positioned at the midclavicular line, situated 2 cm below the costal margin. A comparable triangular arrangement was employed for left-sided cases, with changes in the sizes of the second and third trocars. Dissection commenced with an incision of the white line of Toldt's fascia, and either the ascending or descending colon was reflected medially, contingent upon the side of the nephrectomy, to expose the retroperitoneum. Subsequently, the ureter was delineated and dissected up to the renal hilum for identification of the renal artery and vein, which were ligated using three hem-o-lok clips before transection. Following this, the specimen was extracted upon release of the remaining tissues. In the end, the specimen was completely retrieved within a laparoscopic specimen bag through an extended subcostal port site.

Evaluations and follow-up

Data on demographic information including age, gender, body mass index (BMI), pre-existing medical conditions, submission date, surgical procedure, and discharge particulars were documented. Preoperative assessments were performed, encompassing a full blood count, measurement of serum creatinine and blood urea levels, and evaluation of coagulation profiles. They underwent urinary system ultrasonography, CT, and Tc-99m DTPA renal dynamic scintigraphy to precisely evaluate and diagnose benign, nonfunctioning kidneys.

Operative parameters were recorded, including estimated blood loss, operation time, and conversion to open surgery. During the operation, observations made intraoperatively, including hemorrhage, visceral trauma, and perinephric adhesions, were documented. The extent of adhesion was classified as follows: absence or mild adhesion, denoting instances where separation was easily achievable through blunt dissection; and severe adhesion, indicating dense adhesions necessitating either blunt or sharp dissection for separation. Surgeons underwent a survey to assess their perception of 3D-LN using the Likert scale. Postoperative complications according to the Clavien-Dindo classification and the visual analog scale (VAS) score for pain were assessed. After three months, patients were followed up with a clinical examination and ultrasonography.

Statistical analysis

Data analysis was performed using IBM SPSS Statistics software version 26.0 (IBM Corp., Armonk, NY). Variables are presented as the mean and standard deviations. A p-value of <0.05 was considered to be statistically significant.

## Results

Overall, 40 patients were recruited for our study. Patient demographics and preoperative characteristics are reported in Table 1. The average age was 58.35 ± 14.9. The female:male ratio was 13:27. The chief complaint was flank pain, accounting for 82.5%. In our study, 30% of patients had no history of urological diseases, 45.0% of cases had undergone surgical intervention, and only 25.0% of patients had urological diseases but had not undergone surgical intervention. The mean BMI was 21.04 ± 2.64 kg/m^2^, and the majority of patients had a normal BMI (77.5%). All patients had hydronephrosis based on CT and proved to have a nonfunctioning kidney or estimated GFR <10 mL/min/1.73m^2^ via Tc-99m DTPA renal dynamic scintigraphy. Meanwhile, the contralateral kidney function remained >60 mL/min/1.73m^2^ in 36 cases (90%) and four cases had estimated GFR of the opposite kidney ranging from 41 to less than 60 mL/min/1.73m^2^ (all four patients had estimated GFR of the removed kidney of 0 mL/min/1.73m^2^, meaning that the removed kidney completely lost function).

**Table 1:** Preoperative characteristics eGFR: estimated glomerular filtration rate

Characteristic	Results
Age (years)	58.35 ± 14.9
Gender (male/female)	13/27 (32.5%/67.5%)
Body mass index (kg/m^2^)	21.04 ± 2.64
History	
No urological diseases	12 (30%)
Surgical interventions	18 (45%)
Urological diseases without interventions	10 (25%)
Chief complaint	
Flank pain	33 (82.5%)
Flank pain and fever	6 (15%)
Hypertension	1 (2.5%)
Hydronephrosis grade (based on CT)	
Grade 1	0 (0%)
Grade 2	2 (5%)
Grade 3	20 (50%)
Grade 4	16 (40%)
Renal atrophy	2 (5%)
Renal parenchyma thickness (based on CT)	
<5mm	22 (55%)
≥5mm	18 (45%)
Removed kidney function (%)	
0%	27 (67.5%)
<10%	13 (32.5%)
>10%	0 (0%)
The eGFR of the removed kidney (mL/min/1.73m^2^)	
0	27 (67.5%)
<10	13 (32.5%)
>10	0 (0%)
The eGFR of the contralateral kidney (mL/min/1.73m^2^) <41	0 (0%)
41 - <60	4 (10%)
≥60	36 (90%)

The perioperative clinical indicators of the patients who underwent 3D-LN are shown in Table 2. Seventeen out of 40 patients suffered a right-side nephrectomy, accounting for 42.5%. In our study, the conversion rate to open surgery was 7.5%, which included three cases of severe adhesive kidney, difficult dissection, and inaccessibility of the renal pedicle. The average operative time was 92.57 ± 28.69 minutes, and the mean blood loss was 51.62 ± 24.35 mL. In addition, the difference in surgery time between the group with no adhesion and the group with mild adhesion, as well as between the first 19 patients and the last 18 patients, was statistically significant with p <0.05 (Table 3). In this study, all cases had a benign etiology. The most common cause of urinary stones was 62.5%, followed by ureteral stricture with eight cases (20.0%), ureteral-pelvic junction obstruction accounted for 12.5%, and duplicated ureter and kidney atrophy both had one case. There were two out of 40 patients (5.0%) who had complications during surgery with subcutaneous emphysema.

**Table 2:** Perioperative characteristics 3D-LN: 3D laparoscopic nephrectomy

Characteristic	Results
Location of removed kidney	
Right	17 (42.5%)
Left	23 (57.5%)
Extent of adhesion	
No	19 (47.5%)
Mild	18 (45%)
Severe	3 (7.5%)
Operation time (minutes)	92.57 ± 28.69
≤60	5 (13.51%)
61 – 90	18 (48.65%)
91 – 120	10 (27.03%)
121 – 150	2 (5.41%)
>150	2 (5.41%)
Blood loss (mL)	51.62 ± 24.35
≤30	11 (29.73%)
31 – 60	12 (32.43%)
61 – 90	13 (35.14%)
>90	1 (2.7%)
Perioperative complications	
Subcutaneous emphysema	2 (5%)
Hemorrhage	0 (0%)
Open surgery conversion	3 (7.5%)
Likert scale	
3D-LN increased depth perception	4.48 ± 0.51
3D-LN increased spatial orientation	4.43 ± 0.51
3D-LN caused headache	2.87 ± 1.06
3D-LN caused dizziness	3.48 ± 0.9
3D-LN caused nausea	2.22 ± 1.04
3D-LN caused eyestrain	3.39 ± 0.99
3D-LN satisfaction	4.3 ± 0.7
Future selection of 3D-LN	4.61 ± 0.66
Etiology	
Renal stones	11 (27.5%)
Ureteral stones	14 (35%)
Renal atrophy	1 (2.5%)
Ureteropelvic junction obstruction	5 (12.5%)
Ureteral stricture	8 (20%)
Duplicated ureter	1 (2.5%)

**Table 3:** The relationship between operative time and relative factors

Relative factors		Counts	Operative time (minutes)	p-value
Extent of adhesion	No	19	73.42 ± 11.79	p <0.05
	Mild	18	112.78 ± 27.45	
Learning curve	The first 19 cases	19	105.26 ± 31.91	p <0.05
	The last 18 cases	18	79.17 ± 17.17	

In our study, the majority of surgeons said that the 3D laparoscopy system helps increase depth perception and spatial orientation during surgery. In addition, using the 3D laparoscopic system caused some side effects. Among them, six cases (26.09%) reported headache, 60.87% reported dizziness, three cases reported nausea (13.04%), and an eye strain effect was reported in 13 cases (56.53%).

In terms of postoperative results (Table 4), the average bowel recovery time was 19.59 ± 9.6 hours. The average VAS score was 3.73 ± 1.35, of which 54.05% had no pain or little pain and 45.95% had moderate or high pain. Therefore, there was also a corresponding change in the duration of pain-relief medication use, with 72.97% of patients only using pain-relief medications within the first three days after surgery and 27.03% using pain-relief medications for more than three days. The average time spent using intravenous pain-relief medications was 2.65 ± 1.77 days, and the longest use of the drug was 10 days, which was the case of postoperative pancreatitis.

**Table 4:** Postoperative characteristics VAS: visual analog score

Characteristic	Results
Bowel recovery time (hours)	19.59 ± 9.6
Drainage catheter removal time (hours)	54.97 ± 23.33
VAS score after 24 hours	3.73 ± 1.35
Duration of pain-relief medications (days)	2.65 ± 1.77
Postoperative complications	
Wound infection	1 (2.7%)
Hemorrhage	1 (2.7%)
Pancreatitis	1 (2.7%)
Hospital stay (days)	7.89 ± 3.59
Histology	
Chronic nephritis	23 (57.5%)
Kidney fibrosis	6 (15%)
Glomerulonephritis	9 (22.5%)
Chronic fibrotic interstitial nephritis	2 (5%)

The median length of hospital stay was 7.89 ± 3.59 days. We recorded three cases of postoperative complications, including one case of pancreatitis due to touching the pancreas during dissection, one case of surgical wound infection, and one case of hemorrhage at the base of the drainage tube. In terms of pancreatitis, the patient was treated with Sandostatin. Meanwhile, the hemorrhage case became hemostatic after applying pressure using a cotton ball or gauze. A surgical wound infection was treated with bandages daily and according to an antibiotic chart. All of these complications were treated well and did not threaten the patient. Postoperative follow-up at three months revealed no special clinical symptoms, but there was a case of small seroma at the right renal fossa with a diameter of 0.9 mm.

## Discussion

Transperitoneal nephrectomy was performed first by Clayman in 1990 [2], and one year later, in India, Gaur also had a successful case of retroperitoneal nephrectomy [10]. Current laparoscopic systems have been mainly 2D laparoscopic imaging systems. They had certain limitations, such as a lack of image depth, so it would take a surgeon a long time when performing difficult and complicated movements, such as dissecting the renal pedicle or in cases of severe adhesion and unclear anatomical landmarks. Therefore, the surgery time would be prolonged, making the surgeon tired. To overcome those disadvantages, 3D laparoscopic systems have gradually been widely applied in the past few years and show certain advantages compared to 2D, such as depth perception and spatial orientation [11]. These helped reduce operative time, blood loss, and dissection time, as well as make surgeons more comfortable during surgery [7]. In our study, the subjective feelings of the surgeons showed better depth perception and spatial orientation, especially in complex procedures such as renal pedicle dissection and arteriovenous clamping or cases of severe adhesion.

In terms of operative time, with an average value of 92.57 ± 28.69 minutes, the difference in surgery time between the group with no adhesions and the group with mild adhesions, as well as between the first 19 patients and the last 18 patients, was statistically significant with p <0.05. Masoud et al. compared 40 laparoscopic nephrectomy patients; they were divided into two groups: group I included the first 20 patients, and group II included the last 20 patients. The results showed that the following group of 20 patients had a faster surgery time than the first group of 20 patients (108.5 vs. 139.3 minutes, p <0.05). [12]. Meanwhile, Aminsharifi et al. studied 79 patients who underwent transperitoneal laparoscopic nephrectomy, patients were divided into 29 with and 50 without prior surgery at the same anatomical site. The results indicated that the mean operative time was longer in patients with previous surgery than in the other group (98.6 vs. 62.3 minutes, p = 0.03) [13].

In our study, the average blood loss was 51.62 ± 24.35 mL, and no case required a blood transfusion during surgery. In a study comparing laparoscopic nephrectomy between using 2D and 3D systems, according to Patankar, 2D laparoscopy has a larger blood loss volume than 3D laparoscopy (240 ± 123.16 mL versus 181.43 ± 55.69 mL, p <0.05) [7]. A study by Nguyen et al. also had a similar pattern; the average blood loss in 2D laparoscopic surgery was greater than that in 3D surgery (240.2 ± 211.6 ml versus 160.7 ± 165.3 ml, p <0.05) [11]. The majority of surgeons in our study said that 3D systems helped increase depth perception and spatial orientation during surgery. The study by Agrusa et al. also had similar results; the subjective perception of the surgeon showed that the 3D systems gave better depth perception [14]. Dirie et al. also showed that 3D laparoscopy helped improve the surgeon's depth perception, increased visibility, and gave better clinical and surgical results than 2D laparoscopy [4].

Our conversion rate to open surgery was 7.5%. There were three cases with a history of pyelonephritis managed by a nephrostomy draining the kidney. During the approach, we found that the inflammatory kidney had severe adhesion to the adjacent structures, causing difficulty in dissection and exposure of the renal pedicle during surgery, so we decided to convert it to open nephrectomy. In cases of mild adhesions, we still performed successfully, but when compared with the group without adhesions, the operative time was also longer. Although the kidney has adhesion, dissection is more difficult, better depth perception and spatial orientation of 3D systems make dissection easier than 2D systems.

In our study, three patients had complications after surgery. Among them, one case had pancreatitis (2.7%), because during surgery we touched the pancreas but did not cause gross damage to the pancreas. This patient was treated stably with somatostatin and discharged after 20 days. According to Horesh et al., among 1,674 patients undergoing nephrectomy, there were four patients with pancreatic damage; these four patients had drainage placed to control pancreatic fistulas, and one case required re-operation [15]. Thus, during laparoscopic nephrectomy, we need to pay attention to avoid damaging the pancreas, especially in the left nephrectomy.

We acknowledge that this study had several limitations. The major limitation was that we did not have a control group (2D-LN) to compare the results. Additionally, this study was non-randomized and retrospective. Finally, the postoperative pain intensity and duration until the resumption of normal activities were not assessed due to the retrospective design of this investigation; we used recovery of bowel function and duration of hospital stay to assess the postoperative recovery of patients.

## Conclusions

In this study, we reported our initial experience with the application of 3D surgery. Our results showed that 3D-LN is safe and effective for nonfunctioning kidneys with complications due to benign diseases. Three-dimensional surgery helps increase depth perception and spatial orientation for surgeons and can compensate for the remaining shortcomings of traditional 2D systems. The learning curve typically reaches completion after approximately 20-25 procedures. This insight can aid in the development of training programs aimed at familiarizing new surgeons with this technique.
